# Unsung heroes of the scientific record deserve credit

**DOI:** 10.1038/s41467-018-07336-1

**Published:** 2018-11-20

## Abstract

Reviewers make an essential contribution to scientific progress; *Nature Communications* will now formally acknowledge their role in published articles.

Evaluation of scientific research has come under increased scrutiny recently. Although peer review is widely considered the cornerstone of validating discovery, and there is little doubt that the researchers themselves are best suited to evaluate the work of their peers, the mood is one of change.

Despite ubiquitous apocryphal stories of ‘Reviewer #3’, the principle that peer review improves the scientific record is universally agreed upon. It provides checks, evaluation, and ultimately the seal of approval. But this scrutiny and evaluation take time and effort, and the burden is growing both because more papers are being published by the scientific community and because research is increasingly data-rich and analytically complex. The credibility of the scientific record depends on thorough peer review. Should the invaluable contribution of peer reviewers not be openly acknowledged?

*Peer review should be celebrated for the contribution it makes to shaping the scientific record*

There is considerable interest in opening up the ‘black box’ of peer review, and we have been keenly watching the increasing desire for change in this direction. Since January 2016, *Nature Communications* has been publishing reviewers’ reports alongside scientific papers if the authors choose to do so. Now, 60% of our authors have opted to publish the comments made on their manuscripts. Making reviewers’ comments available alongside the research they helped shape has a number of benefits, not least aiding transparency of the publication process or providing training material for early career researchers.

While opinions are divided on whether reviewers’ reports should remain anonymous, there is a growing call for greater recognition of the contribution of reviewers. And rightly so! Being a reviewer, let alone a good, constructive reviewer, has unfortunately always been a peripheral consideration when it comes to promotion or funding applications. The emphasis has consistently been almost entirely on the research output in the form of authorship of scientific papers, even though being a scientist entails so much more.

*Nature* recently started a trial in which reviewers can be acknowledged by name in the publications they have helped shape. We are delighted to announce that we are now introducing this to *Nature Communications*. From today, reviewers will be asked when submitting their comments on a manuscript under consideration at *Nature Communications* if they would like to be named on the paper, should it be published. We will also be encouraging them to sign their comments to the authors, with the aim that, if the comments are published, they will receive full credit for their contribution to the work. Should a reviewer opt out of being named, we will acknowledge their contribution anonymously.

Peer review should be celebrated for the contribution it makes to shaping the scientific record. Being a reviewer entails a great deal of effort and responsibility, but often the anonymity of the peer review process prevents the appropriate attribution of credit and prestige. Increasingly, publishers and third-party services are finding ways to recognise the contribution of individual reviewers while abiding by the confidentiality policies of the journals, or lifting the anonymity veil altogether. At Nature Communications, we are keen to do our part in supporting this trend.

We are operating this as a trial and will report back on its success, and its operation with our existing transparent peer review scheme, within a year. We will also be collecting demographic information, on a voluntary basis, such as gender, country of affiliation, and career stage, from our reviewers so that we can meaningfully assess the impact and level of uptake of this trial. Further details can be found in a dedicated FAQ document.

